# Solution‐Processed Faraday Rotators Using Single Crystal Lead Halide Perovskites

**DOI:** 10.1002/advs.201902950

**Published:** 2020-02-13

**Authors:** Randy P Sabatini, Chwenhaw Liao, Stefano Bernardi, Wenxin Mao, Matthew S. Rahme, Asaph Widmer‐Cooper, Udo Bach, Shujuan Huang, Anita W. Y. Ho‐Baillie, Girish Lakhwani

**Affiliations:** ^1^ ARC Centre of Excellence in Exciton Science School of Chemistry The University of Sydney Sydney NSW 2006 Australia; ^2^ Australian Centre for Advanced Photovoltaics (ACAP) School of Photovoltaic and Renewable Engineering University of New South Wales Sydney 2052 Australia; ^3^ ARC Centre of Excellence in Exciton Science Department of Chemical Engineering Monash University Clayton VIC 3800 Australia; ^4^ School of Engineering Macquarie University Sydney NSW 2109 Australia; ^5^ School of Physics University of Sydney Nanoscience Institute The University of Sydney Sydney NSW 2006 Australia

**Keywords:** Faraday rotation, magneto‐optics, methylammonium lead bromide, optical rotation, perovskites, single crystals, terbium gallium garnet, Verdet constant

## Abstract

Lead halide perovskites (LHPs) have become a promising alternative for a wide range of optoelectronic devices, thanks to their solution‐processability and impressive optical and electrical properties. More recently, LHPs have been investigated in magneto‐optic studies and have exhibited spin‐polarized emission, photoinduced magnetization, and long spin lifetimes. Here, the viability of methylammonium lead bromide (MAPbBr_3_) single crystals as solution‐processed Faraday rotators is demonstrated. Compared to terbium gallium garnet, the industry standard in the visible, it is found that MAPbBr_3_ exhibits Verdet constants (i.e., strength of Faraday effect) of similar or greater magnitude (up to 2.5x higher), with lower temperature dependence. Due to its low trap absorption, it is calculated that an optical isolator made from MAPbBr_3_, with appropriate antireflection coatings, should reach ≈95% transmission and achieve 40 dB isolation for incoming powers of over 2 W. It is also shown that the Verdet constant of MAPbBr_3_ can be calculated accurately from its dispersion in refractive index, allowing the possibility to predict similar effects in other perovskite materials.

## Graphical abstract

Strong Faraday rotation is demonstrated for methylammonium lead bromide (MAPbBr_3_) single crystals. Coupled with low defect density, they are poised to represent a solution‐processed alternative for Faraday rotator applications, such as optical isolators and optical switches. Additionally, their Faraday rotation fits well with classical theory, allowing prediction of other perovskites for this application.

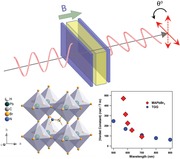

## 1 Introduction

**Figure 1:**
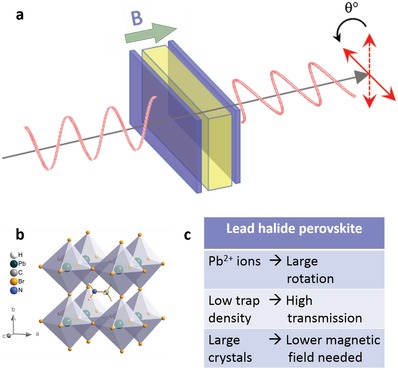
Perovskites as Faraday rotators. a) Schematic of Faraday rotation. Polarization of incoming light is rotated by the medium within a magnetic field. b) Single crystal structure of MAPbBr_3_. c) Attributes of LHPs that make them desirable as Faraday rotators.

During the last decade, lead halide perovskites (LHPs) have become promising materials for a range of optoelectronic applications, such as photovoltaics,^1^, ^2^ light‐emitting diodes,^3^, ^4^, ^5^ lasers,^6^, ^7^, ^8^ and photodetectors.^9^, ^10^ As solution‐processed materials, perovskites pose an attractive alternative to traditional silicon and III‐V semiconductors, which are typically grown by more demanding Czochralski (Cz),^11^ vertical gradient freeze,^12^ or molecular beam epitaxy^13^ methods. More recently, LHPs have been investigated for their magnetic properties, including spin polarized luminescence,^14^, ^15^, ^16^ photo‐induced magnetization,^17^ and long spin lifetime.^16^, ^18^, ^19^ Based on their impressive magneto‐optic properties, we sought to pursue their application as a Faraday rotator (**Figure** **1**a).

Faraday rotators are magneto‐optic devices that rotate the polarization of transmitted light in a nonreciprocal fashion (i.e., same direction of rotation for both forward and backward light propagation).^20^ These properties have made Faraday rotators the primary component in optical isolators,^21^ as well as optical circulators^22^ and Faraday mirrors.^23^ The Verdet constant (V), given in units of rad (T m)^−1^, is a measure of how efficiently a material rotates the polarization of light,^20^ with the figure of merit (FOM) often defined as the Verdet constant divided by the absorption coefficient (α).^24^ The current industry standard for the visible region is terbium gallium garnet (TGG);^25^ however, TGG is fabricated by the Czochralski method,^26^ which requires high heat (≈1500 °C) and inert conditions (quartz chamber in argon atmosphere), thus increasing the cost of crystal production.

A solution‐processed material would lower both the cost and complexity of production. However, for even low‐to‐moderate power applications, this material would need to exhibit a high Verdet constant, low trap absorption, and large crystal growth. LHPs (Figure 1b) have been demonstrated previously to exhibit very small trap densities,^27^, ^28^ and their synthesis has been developed to yield large crystals.^29^, ^30^ In addition, for diamagnetic glasses, the largest visible Faraday rotations come from those doped with Tl^+^, Pb^2+,^ and Bi^3+^, whose large atomic numbers and radii lead to large magnetizations.^24^ Recently, a diamagnetic (1‐*x*)GeS_2_‐In_2_S_3_‐(*x*)PbI_2_ chalcogenide glass was shown to have a Verdet constant of ≈83 rad (T m)^−1^, due to the added PbI_2_.^31^ We thus hypothesized that a LHP single crystal would exhibit an even higher Verdet constant (Figure 1c).

Herein we demonstrate that single crystal methylammonium lead bromide (MAPbBr_3_) perovskite exhibits Verdet constants up to 470 rad (T m)^−1^. Between 570 and 700 nm, we find that MaPbBr_3_ has Verdet constants comparable to or higher than TGG, with less temperature dependence. While MAPbBr_3_ has a lower FOM than TGG, we calculate that an optical isolator made from MAPbBr_3_, with the appropriate antireflection coatings, should still reach ≈95% transmission and achieve 40 dB isolation for incoming powers of over 2 W. In addition, the solution‐processability of perovskite represents great potential going forward.

## 2 Results and Discussion

We began by growing methylammonium lead bromide (MAPbBr_3_) single crystals by both the inverse temperature crystallization^30^ and antisolvent^32^ method. This produced single crystals with thicknesses ranging from 1 to 4 mm (Figure S1a, Supporting Information). The powder x‐ray diffraction pattern (PXRD) is consistent with simulations (Figure S1b, Supporting Information), and the single crystal XRD (SCXRD) reveals the typical cubic crystal structure of MAPbBr_3_ (Figure 1b, Table S1, Supporting Information).

**Figure 2:**
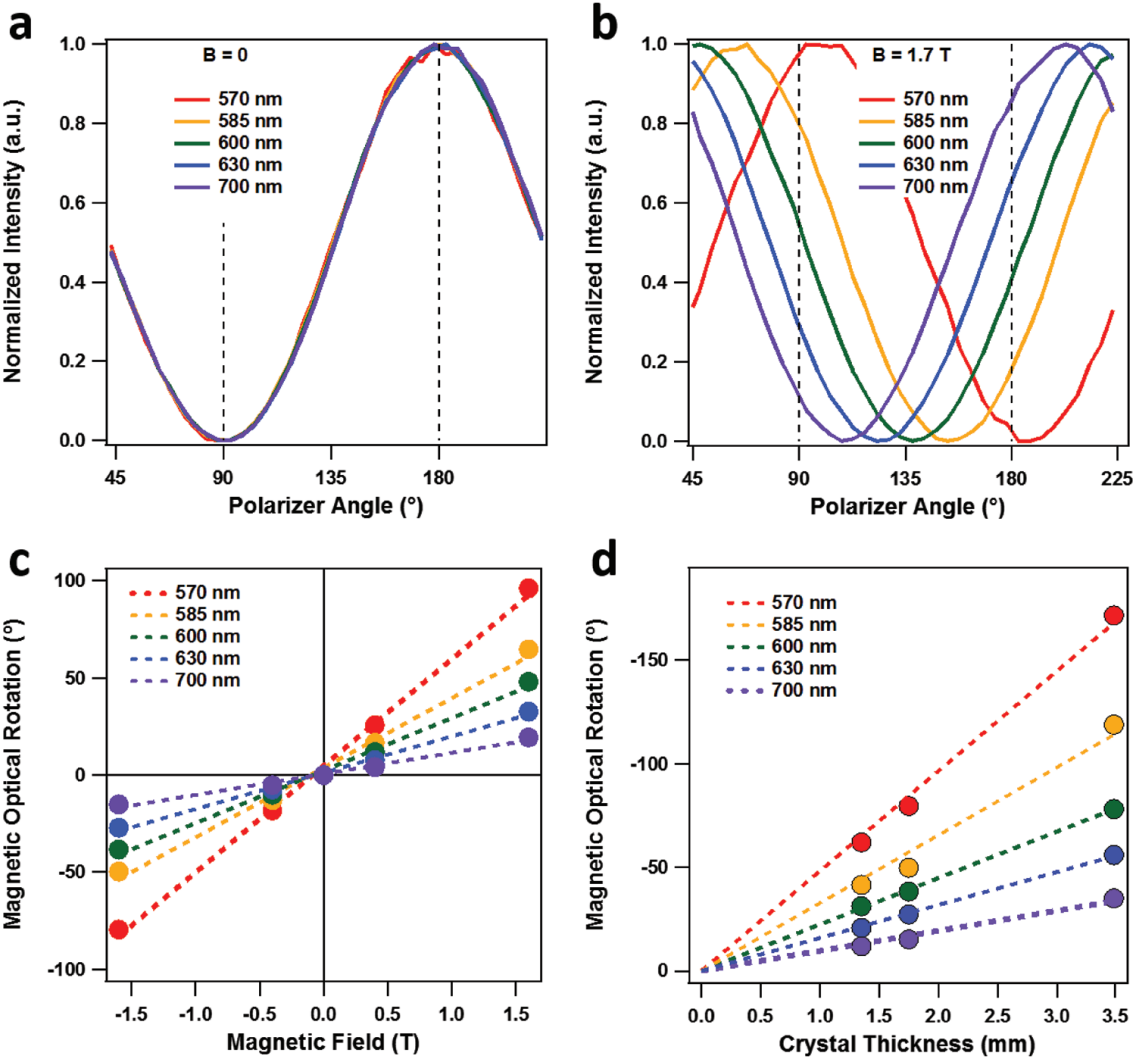
Effects of magnetic field and crystal thickness. a) Normalized transmission intensity versus polarizer angle for a 1.75 mm crystal, for different wavelengths in the absence of a magnetic field (*B* = 0 T). b) Normalized transmission intensity versus polarizer angle for a 1.75 mm crystal, for different wavelengths in a 1.7 T magnetic field. c) MOR for a 1.75 mm crystal as a function of magnetic field for different wavelengths. d) MOR as a function of crystal thickness for different wavelengths in a −1.7 T magnetic field.

Testing for optical rotation was performed in a home‐built setup (Figure S2a, Supporting Information). A white‐light laser is sent through a polarizer to select an initial polarization, while the second polarizer acts as an analyzer, detecting any change in the polarization induced by the material within the magnetic field. In the absence of a magnetic field, the MAPbBr_3_ crystal imparts only a small optical rotation to the light polarization (< 2° mm^−1^). Thus, the shape of the transmission spectrum remains the same, and the signal for all wavelengths is maximized when the angle of the two polarizers are roughly parallel (**Figure** **2**a, S2b, Supporting Information). However, when the MAPbBr_3_ crystal is placed within a magnetic field, it imparts a large rotation that is wavelength dependent, thus distorting the shape of the transmission spectrum (Figure S2c–d, Supporting Information). Essentially, this means that different wavelengths will have different θ_min_ and θ_max_ values (Figure 2b). As expected, the direction of optical rotation switches sign based on the direction of the magnetic field (i.e., N→S or S→N) (Figure 2c).

The angle of magnetic optical rotation (MOR) is given by

(1) MOR = VBL

where *V* is the Verdet constant, *B* is the magnetic field, and *L* is the thickness of the crystal. To demonstrate the Faraday effect thoroughly, we have measured MOR at four different magnetic fields (−1.7, −0.46, 0.46, and 1.7 T), as well as three different crystal thicknesses (1.35, 1.75, and 3.48 mm). We observe a linear trend for both parameters (Figures 2c–d), consistent with theory. The MOR exhibits a clear dependence on wavelength, with a greater degree of rotation at wavelengths closer to the bandgap.

**Figure 3:**
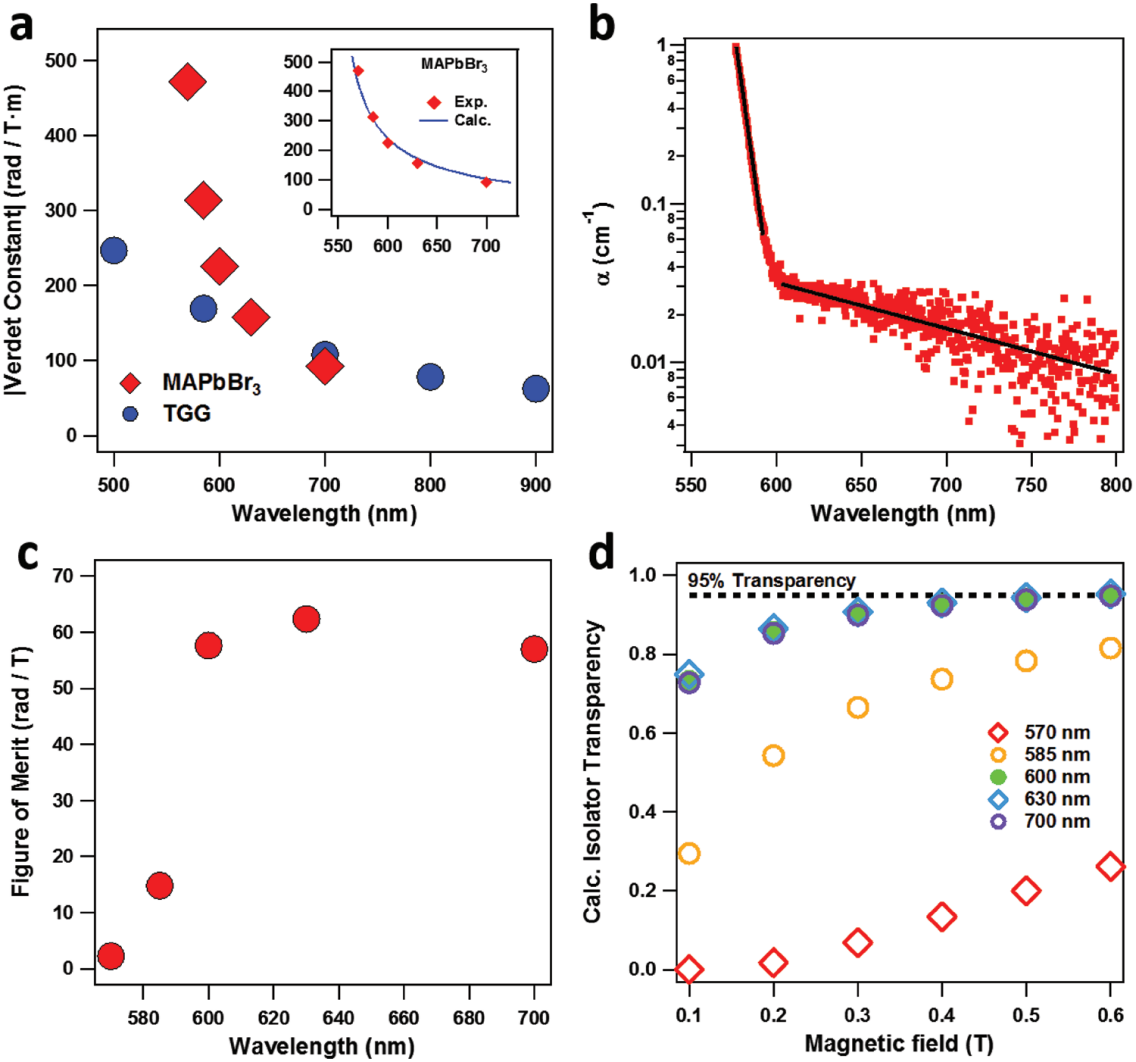
Metrics of MAPbBr_3_ Faraday rotation. a) Wavelength dependence of the Verdet constant (absolute values) for single crystals of MAPbBr_3_ and TGG control. (inset) Comparison of the experimental Verdet constants at different wavelengths for MAPbBr_3_ single crystal and values predicted from the change in refractive index (Becquerel formula). b) Absorption coefficient of MAPbBr_3_ single crystal. c) Wavelength dependence of figure of merit for MAPbBr_3_ single crystal. d) For different magnetic fields, calculated transparency of an MAPbBr_3_‐based optical isolator at select wavelengths, assuming appropriate antireflection coating.

From these measurements, the Verdet constant can be determined for each crystal (Figure S3a–c, Supporting Information), and an average value (with error) can be calculated (Figure S3d, Supporting Information, **Table** **1**). These data show the wavelength dependence of the Verdet constant for MAPbBr_3_, fit to the single oscillator model:

(2) $$V\;\left(\lambda \right)\;=\;\frac{A}{\lambda^{2}-\lambda_{0}^{2}}$$

where λ_0_ corresponds to the bandgap transition, and *A* is a constant proportional to the magnetization and transition probability.^24^ We have also tested TGG as a control and measured its corresponding trend of optical rotation with magnetic field (Figure S4, Supporting Information). As a paramagnetic material, TGG exhibits a negative Verdet constant, whereas the diamagnetic MAPbBr_3_ exhibits a positive one.^33^ For comparison, we plot the absolute values of their Verdet constants for different wavelengths (**Figure** **3**a). The Verdet constant of MAPbBr_3_ reaches values of up to 470 rad (T m)^−1^ at 570 nm. This is ≈2.5x higher than that of TGG at the same wavelength, and the Verdet constant of MAPbBr_3_ remains higher until ≈650 nm. While the slope of MAPbBr_3_'s Verdet constant is steeper (due to a lower energy λ_0_, Figures S3–4, Supporting Information), limiting its functional wavelength range, perovskites of other composition can be fabricated to expand to other wavelengths (e.g., through use of different halides).

**Table 1:** Metrics of MAPbBr_3_ for selected wavelengths

λ (nm)	V (rad T^−1^ m^−1^)	α (cm^−1^)	(1/V) (dV/dT) (10^−3^ K^−1^)	FOM (rad T^−1^)
570	471	2.096	−2.3^a)^	2.2
585	314	0.212	−4.1	14.8
600	226	0.039	−2.9	57.6
630	157	0.025	−1.7	62.4
700	92	0.016	−1.0	57.0

Abbreviation: V, Verdet constant; α, absorption coefficient; (1/V) (dV/dT), variation of Verdet constant with temperature; FOM, figure of merit

^a)^ Temperature dependence of 570 nm does not follow trend, which we attribute to its close proximity to the bandgap.

The high Verdet constant of MAPbBr_3_ can be attributed to several properties of LHPs. Owing to its large atomic number and ionic radii, the Pb^2+^ cation is highly polarizable,^34^ which indicates a large diamagnetic magnetization.^35^ As a whole, MAPbBr_3_ is also well‐known for its large absorption coefficient.^28^ These features have been shown to lead to high Verdet constants,^24^ effectively increasing the *A* coefficient in Equation (2). Additionally, the transition of MAPbBr_3_ lies in the visible region. As the Verdet constant is highest at wavelengths close to λ_0_, this enables increased performance in the visible. While this effect is similar to that of lead‐doped glasses, the high density of Pb^2+^ in the perovskite lattice improves greatly the Verdet constant.^36^

For a deeper understanding, we set out to calculate the Verdet constant of MAPbBr_3_ from theory. While the Faraday effect in paramagnetic materials (such as TGG) requires quantum analysis,^37^ it can be treated classically in diamagnetic materials. Here we apply the Becquerel formula:^38^

(3) $$V\;=\;\frac{\gamma e}{2m_{\text{e}}c}\;\lambda \frac{dn}{d\lambda }$$

where *e* and *m*_e_ are the elementary charge and mass of an electron, respectively, *c* is the speed of light in a vacuum, λ is wavelength, and $\frac{dn}{d\lambda }$ is the wavelength dispersion of the refractive index. This formula often contains the term, γ, called the magneto‐optic anomaly, which is a multiplicative factor meant to quantify disagreement between calculated and experimental values.^39^ It was rationalized to describe the degree of covalent bonding in a crystal, where for pure ionic crystals γ = 1.^40^ In this equation, the influence of polarizability/absorption are incorporated into the change in refractive index, which can be related to polarizability via the Lorentz–Lorentz equation^41^ or absorption via the Kramers–Kronig transformation.^42^

To calculate the Verdet constant, we fit literature values^43^ for the refractive index using the Sellmeier equation to obtain the $\frac{dn}{d\lambda }$ term (Figure S5, Supporting Information). The Verdet constants at different wavelengths were then calculated and compared with those obtained experimentally (Figure 3a inset). For MAPbBr_3_, the Becquerel formula predicts accurately not only the wavelength dispersion of the Verdet constant but also the magnitude, without the need for the multiplicative factor (i.e., γ = 1); we attribute this to the strong ionic character of MAPbBr_3_. As a class, LHPs encompass a wide range of materials, spanning different cations, halides, and dimensionalities. The excellent fit here for MAPbBr_3_ suggests that the refractive indices of these materials may prove useful as a predictive guide toward alternative perovskite materials with high Verdet constants.

Absorption loss is another important metric for Faraday rotators. To this end, we have measured the sub‐bandgap absorption spectrum of a 3.48 mm crystal in an integrating sphere (Figure 3b, Figure S6, Supporting Information), finding absorption coefficients ranging from 2 cm^−1^ for 570 nm to 0.016 cm^−1^ for 700 nm (Table 1). These numbers are roughly consistent with what has been shown previously.^28^ Owing to the low defect density in perovskite single crystals, the trap absorption is small for solution‐processed materials. However, it does fall short of what is obtained for TGG. While our FOM reaches ≈62 rad T^−1^ (Figure 3c, Table 1), similar to that of ceramic TGG,^44^ it is smaller than that of single crystal TGG, whose FOMs in the literature range from 150 to ≈1000 rad T^−1^ in this region.^45^, ^46^ Nevertheless, based on our FOMs, we calculate that an MAPbBr_3_‐based optical isolator (made for 45° rotation) could reach ≈95% transparency with magnetic fields of ≈0.5 T for 600, 630, and 700 nm (Figure 3d), assuming appropriate anti‐reflection coating (Note S1, Supporting Information). Continual improvement in either the defect density or Verdet constant would allow higher transparency at lower magnetic fields or for a wider wavelength range.

**Figure 4:**
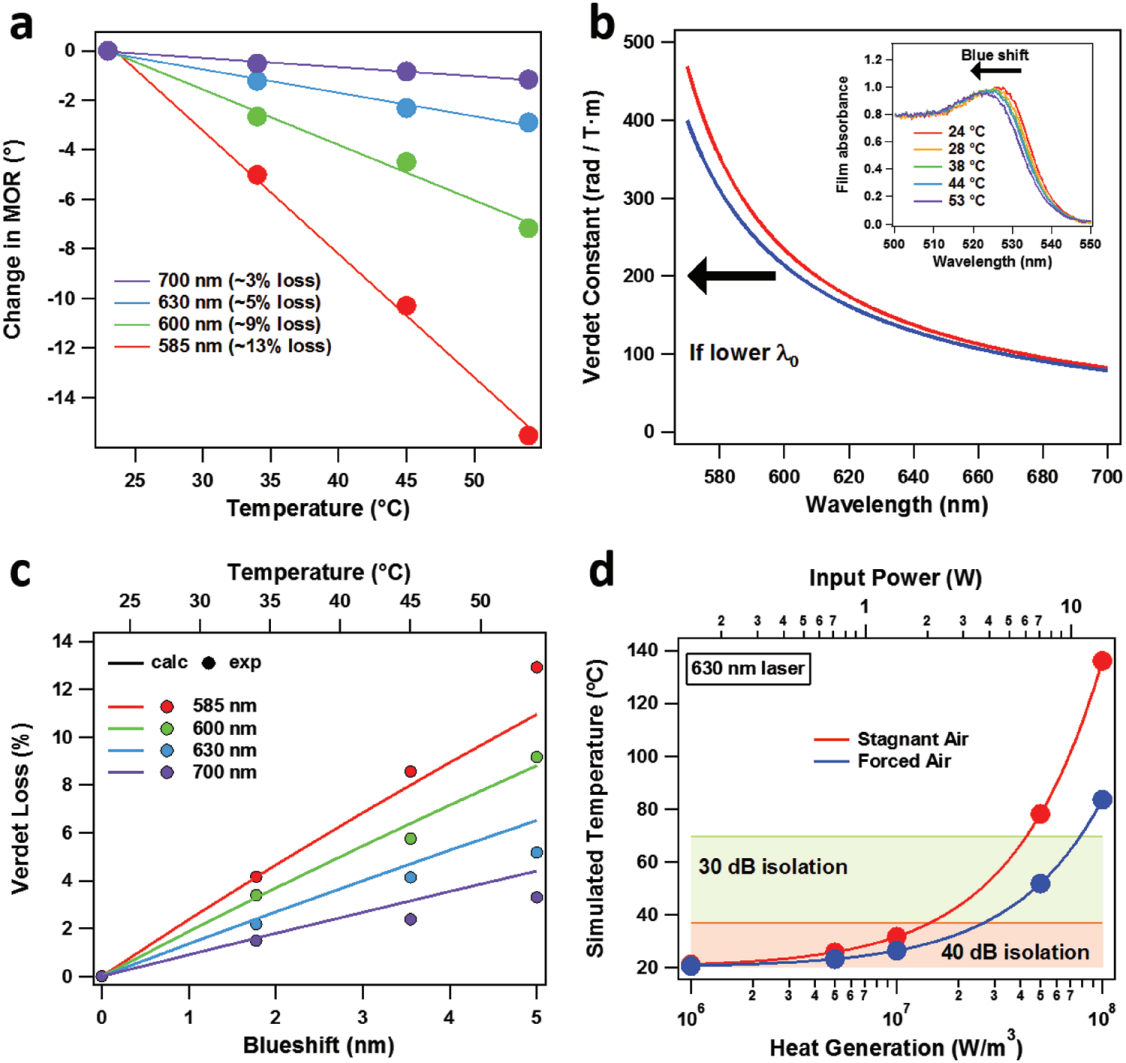
Temperature dependence. a) Decrease of total optical rotation for MAPbBr_3_ single crystal. b) Wavelength dispersion of Verdet constant as a function of blue‐shifting λ_0_ values. Inset: Blue‐shifting of an MAPbBr_3_ film as a function of temperature. c) Calculated loss (%) of Verdet constant as a function of blue‐shifting. d) Thermal simulations for MAPbBr_3_ single crystal for given 630 nm input powers (1 mm spot diameter) in the presence of stagnant and forced air. Temperatures are shaded for which 30 and 40 dB isolation would be achievable in an optical isolator.

We then investigated the effect of temperature on the Verdet constant. Paramagnetic materials, such as TGG, are known to vary strongly with inverse temperature $\left(V_{\text{par}}\propto \;\frac{1}{T}\right)$, as their Verdet constants are proportional to their temperature‐dependent paramagnetic susceptibility.^24^ Near room temperature, TGG has been shown to exhibit a temperature dependence of $\frac{1}{V}\frac{\partial V}{\partial T}$ = 3.47 × 10^−3^ K^−1^, which was nearly wavelength‐independent.^47^ In contrast, the Verdet constants of diamagnetic materials, such as MAPbBr_3_, should in principle be unaffected by temperature.^24^ However, we find that the Verdet constant of MAPbBr_3_ does decrease with increasing temperature, although unlike for TGG, this effect is proportional to T (versus T^−1^) and becomes less pronounced at longer wavelengths (**Figure** **4**a, Table 1).

As $V\;(\lambda )=\frac{A}{\lambda^{2}-\lambda_{0}^{2}}$, the Verdet constant will decrease if either *A* or λ_0_ decreases. If *A* were to decrease, we would expect that the Verdet constant would lower by the same percentage across the entire spectrum (Figure S7, Supporting Information), which does not fit our experimental data. However, if λ_0_ were to decrease, the Verdet constant would lower by the greatest percentage at bluer wavelengths, as its slope is steepest close to the bandgap (Figure 4b). For reference, we have measured linear absorption spectra of an MAPbBr_3_ thin film at elevated temperatures. Consistent with literature,^48^, ^49^ we observe a blueshift in the exciton peak (Figure 4b, inset). We can then calculate the expected decrease in Verdet constant for different degrees of blue‐shifting, leaving *A* constant. We find that we can reproduce the losses for wavelengths 585–700 nm by estimating a 5 nm blueshift (λ_0_ = 538.4 → 534.4 nm) in λ_0_ (Figure 4c). Note that the losses at 570 nm do not follow this trend (*V*_loss − 570_ ≈ 7%); we attribute this to its close proximity to the bandgap, where other factors may be present.

As with other applications, stability is a key factor to discuss. Based on the crystals studied, we find that the Verdet constants of newly made single crystals are no different from those sitting in air for over 6 months or under continual optical pumping (Figure S8, Supporting Information). While perovskites have traditionally suffered from thermal and photodegradation, the literature has shown that MAPbBr_3_ single crystals are relatively stable.^29^ A previous study has employed thermogravimetric analysis on single crystal MAPbBr_3_ under nitrogen and found that thermal decomposition begins at 320 °C.^50^ Additionally, Raman shifts in an MAPbBr_3_ crystal were shown to be unchanged for 30 days, unencapsulated in ambient conditions.^51^ An MAPbBr_3_ film was also found to be moisture‐insensitive, as no change in either film color or XRD spectrum was observed after being left in ambient atmosphere for 5 months at humidity levels reaching ≈70%.^52^ For our application, we expect photostability to be aided by the sub‐bandgap energy of the photons, which will only be absorbed by trap states.

However, since trap absorption would result in rising temperatures in Faraday rotators/isolators, we next performed thermal simulations to explore how performance would be affected (see Table S2 for input parameters, Supporting Information). We envisioned the MAPbBr_3_ crystal surrounded by a copper heatsink on four sides (Figure S9a, Supporting Information). The pathlength for the crystal was chosen to be 16 mm, the pathlength required for 45° rotation of 630 nm light; control of lateral dimensions and thicknesses have been demonstrated for perovskite single crystals in literature.^30^, ^53^ The other dimensions, 16 and 4 mm, were chosen based on typical aspect ratios for large perovskite crystals.^29^ The excitation spot size was set to 1 mm in diameter, a common aperture size for single frequency lasers used in Raman spectroscopy. For 630 nm pumping, we simulate the temperature increase based on the absorption coefficient at this wavelength, assuming all absorbed photons dissipate non‐radiatively as heat. To remove heat from the system, we incorporate air convection (Figure S9b, Supporting Information) ranging from 5 W m^−2^ C^−1^ (stagnant air) to 50 W m^−2^ C^−1^ (forced air).^54^ Heat generation/input powers (Figures S9c–d, Supporting Information) range from 1 × 10^6^–1 × 10^8^ W m^−3^ / 140 × 10^−3^ W–14 W.

Upon heating, an initial temperature increase coincides with transferring heat from the substrate to the copper heat sink, with the second increase associated with heat removal from convection; after this, equilibrium is reached. We show the final equilibrated temperature for a given heat generation/input power for both stagnant and forced air convection (Figure 4d). This increase in temperature can limit performance of the Faraday rotator as an optical isolator; for example, to maintain isolation of 30 dB, the angle of rotation can only change by ≈3.6°, while 40 dB isolation only allows a 1.14° change.^47^ Inputting the value obtained from the temperature‐dependent measurements, we can estimate the highest input power for which 40 dB isolation is achievable. When convection proceeds through stagnant air, powers over 2 W are possible. This number rises to almost 4 W if convection is through forced air.

## 3 Conclusion

In summary, we have demonstrated the potential of MAPbBr_3_ as a solution‐processed alternative to the current industry standard Faraday rotator, TGG. Between 570 and 700 nm, MAPbBr_3_ exhibits Verdet constants comparable to or greater than (up to 2.5x higher) that of TGG, with lower temperature dependence. Due to low trap absorption, we calculate that an optical isolator made from MAPbBr_3_, with the appropriate antireflection coatings, should reach ≈95% transmission and achieve 40 dB isolation for incoming powers of over 2 W. We also show that the Verdet constant of MAPbBr_3_ can be calculated accurately from its dispersion in refractive index, allowing the possibility to predict similar effects in other perovskite materials.

## 4 Experimental Section

### 4.1 Chemicals and Reagents

CH_3_NH_3_Br (GreatCell Solar), PbBr_2_ (Alfa Aesar 99.999%), and N,N‐dimethylformamide (anhydrous DMF, Alfa Aesar ≥99.8%)%), and dichloromethane (DCM, Alfa Aesar 99.7%) are of reagent grade quality and were obtained from commercial sources without further purification. TGG single crystal <111> was bought from Qunics.

### 4.2 Preparation of CH_3_NH_3_PbBr_3_ Single Crystals by Inverse Temperature Crystallization Method

1.0 m of CH_3_NH_3_PbBr_3_ perovskite precursor solution was prepared by dissolving equimolar amounts of CH_3_NH_3_Br (0.45 g) and PbBr_2_ (1.47 g) in 4 mL DMF at 50 °C by stirring overnight. The solution was filtered with a PTEE filter (0.45 × 10^−6^ m pore size). A small glass vial was filled with 4 mL filtered solution and kept in an oil bath. The solution was heated to 65 °C and this temperature was maintained for 3–5 h for single crystal growth. All the procedures were operated under ambient conditions.

### 4.3 Preparation of CH_3_NH_3_PbBr_3_ Single Crystals by Anti‐Solvent Vapor‐Assisted Crystallization Method

CH_3_NH_3_PbBr_3_ perovskite precursor solution was prepared by dissolving 0.8 M CH_3_NH_3_Br (0.36 g) and 0.64 M PbBr_2_ (0.94 g) in 4 mL DMF at 50 °C by stirring overnight. The solution was filtered with a PTEE filter (0.45 × 10^−6^ m pore size). A small glass vial was filled with 4 mL filtered solution and sealed with foil which had small pinholes to allow DCM vapor to dissolve slowly into perovskite precursor solution. DCM was used as an anti‐solvent to precipitate the single crystals. The glass vial was stored under DCM atmosphere, and the CH_3_NH_3_PbBr_3_ single crystals grew continuously for two days. All the procedures were operated under ambient conditions.

### 4.4 Fabrication of CH_3_NH_3_PbBr_3_ Perovskite Thin Film

Thick glass substrates (1.2 mm) were cleaned with 2% Hellmanex, acetone, and 2‐propanol for 20 min, sequentially. After drying, the substrate was treated with UV ozone for 20 min. The 1 m CH_3_NH_3_PbBr_3_ precursor solution was prepared by dissolving equimolar amounts of CH_3_NH_3_Br (0.11 g) and PbBr_2_ (0.37 g) in 1 mL DMF. The precursor solution was deposited on a cleaned substrate which was spun at 2000 rpm and 6000 rpm for 15 s and 30 s, respectively. During the last 20 s of the second spin‐coating step, the anti‐solvent chlorobenzene was drop‐casted for 3 s. The films were then annealed at 80 °C for 15 min on a hot plate.

### 4.5 Measurement of Crystal Thickness

Thicknesses of crystals were measured with a digital calliper (Mitutoyo).

### 4.6 X‐ray Diffraction

The single crystalline structure was determined with a Bruker Kappa Apex diffractometer by using micro‐focus Mo‐Kα radiation (λ = 0.71073 Å) at 123 K. The structure was solved by direct methods and refined on F^2^ by full‐matrix least‐squares using SHELXTL. The powder XRD data was recorded on an Xpert Multipurpose XRD (PANalytical), with a Cu‐Kα_1_ source at room temperature.

### 4.7 Magnetic Optical Rotations Experiments

Measurements were carried out on a home‐built setup. A white light laser source (NKT Photonics, Compact) was sent through a 550 nm long‐pass filter (to avoid possible complications from excessive absorption above the bandgap). The light was then passed through a Glan–Thompson polarizer (Thorlabs). The sample consisted of a crystal attached to a glass slide using transparent double‐sided tape. The sample was either held in open air (0 T) or inside stationary magnets (±1.7 or ±0.46T) (Jasco). The magnetic field of the permanent magnets was checked with a Gaussmeter (LakeShore, 475 DSP). After passing through the sample/magnet, the light was then passed through a second Glan–Thompson polarizer (Thorlabs) before entering a mini integration sphere (Thorlabs), fiber‐coupled into a spectrometer (Ocean Optics, Flame). The orientation of the second polarizer was adjusted to analyze the degree of rotation.

For temperature‐dependent measurements, the MAPbBr_3_ sample was placed inside a 5 mm pathlength cuvette, which was filled with glycerol and placed in the magnet. A variable‐temperature heat gun (Ozito) was used to heat the sample, and a thermocouple inside the glycerol was used to measure the change in temperature.

### 4.8 Absorption Measurements

Absorption measurements were carried out in an integrating sphere taken from a time‐gated fluorescence lifetime setup (PTI). Crystals were attached to glass sides using transparent double‐sided tape, and a lens was used to focus the light onto the crystal (≈30° angle). Spectra were measured with incoming light in direct and indirect contact with the crystal. A lens was used to couple the outgoing emission into a fiber, which then relayed the light into a spectrometer (Ocean Optics, Flame). See Figure S6 (Supporting Information) for additional information.

### 4.9 Calculations for Isolator Transparency

For each magnetic field strength, the crystal thickness required to achieve 45° optical rotation was calculated, based on MOR = VBL. From these numbers, the isolator transparency was calculated based on the absorption coefficient at each wavelength. These calculations assume lossless antireflection coating.

### 4.10 Thermal Simulations

Thermal simulations were run with the Ansys simulation software package, modelling the transient thermal response under continuous excitation. The thickness of the crystal (16 mm) was chosen as this thickness would result in 45° rotation based on its Verdet constant at 630 nm. The other dimensions (16 and 4 mm) were chosen based on approximate aspect ratios observed for large crystals.^29^ The chosen spot size was 1 mm in diameter, similar to the aperture diameter of single frequency lasers used in Raman spectroscopy. Input powers were translated into heat generation using the absorption coefficient of our crystal, assuming all sub‐bandgap absorption leads solely to heat formation. Heat was removed from the system using air convection, either using stagnant air (5 W m^−2^ C^−1^) or forced cooling (10–50 W m^−2^ C^−1^).^54^ Simulations were run until temperatures approached equilibrium. Parameters of MAPbBr3 for thermal simulations were taken from literature.^55^, ^56^, ^57^ See Table S2 (Supporting Information) for more information.

### 4.11 Calculations of Isolation Thresholds

Extinction ratios (ER) were calculated for changes in optical rotation from the standard 45°, based on ER = [1 + sin(2θ)/[1 + sin (2θ)].^47^ For reference, ER of 1000 and 10 000 are equivalent to 30 and 40 dB isolation, respectively. For a given ER, the change in optical rotation was converted into a percentage lost. For example, ER = 1000 corresponds to a change of ≈± 1.8°, for a total of 3.6° (46.8°–43.2°); this allows a total of ≈8% loss in rotation. From the fits in Figure 4c, a given temperature increase relates to a percent decrease of Verdet constant. As the Verdet constant is proportional to optical rotation, the thresholds were then found.

## Conflict of Interest

The authors declare no conflict of interest.

## Author Contributions

R.S. and G.L. conceived the study. R.S. performed the magneto‐optical experiments, with the help from M.R. for the temperature‐dependent measurements. C.L. and W.M. synthesized the perovskite single crystals. C.L. performed the PXRD and SCXRD. R.S. and S.B. performed the thermal simulations. S.B. performed the refractive index and Verdet constant modelling. G.L. supervised the project, with help from A.W.C., U.B., S.H., and A.W.Y.H.B. R.S., S.B., and G.L. wrote the manuscript. All authors read and commented on the manuscript.

## Supporting information

Supporting information
