# Supplementary material for: Solution‐Processed Faraday Rotators Using Single Crystal Lead Halide Perovskites
Source: Adv Sci (Weinh). 2020 Feb 13;7(7):1902950. doi: 10.1002/advs.201902950 (PMC7141042; doi:10.1002/advs.201902950)
Supplement: Supplementary file 1 — Supporting information [file ADVS-7-1902950-s001.pdf]

Copyright WILEY-VCH Verlag GmbH & Co. KGaA, 69469 Weinheim, Germany, 2020.

Supporting Information

for *Adv. Sci.,* DOI: 10.1002/advs.201902950

Solution-Processed Faraday Rotators Using Single Crystal Lead Halide Perovskites

*Randy P Sabatini, Chwenhaw Liao, Stefano Bernardi, Wenxin Mao, Matthew S. Rahme, Asaph Widmer-Cooper, Udo Bach, Shujuan Huang, Anita W. Y. Ho-Baillie, and Girish Lakhwani**

Supporting Information

Solution-processed Faraday rotators using single crystal lead halide perovskites

Randy P Sabatini[1], *Chwenhaw Liao*[2], *Stefano Bernardi*[1], *Wenxin Mao*[3]; *Matthew S. Rahme*[1], *Asaph Widmer-Cooper*[1], *Udo Bach*[3], *Shujuan Huang*[2,4], *Anita W.Y. Ho-Baillie*[2,4], *Girish Lakhwani*[1]*

[1]ARC Centre of Excellence in Exciton Science, School of Chemistry, University of Sydney, NSW 2006, Australia

[2]School of Photovoltaic and Renewable Energy Engineering, UNSW, NSW 2052, Australia

[3]ARC Centre of Excellence in Exciton Science, Department of Chemical Engineering, Monash University, VIC, 3800, Australia

[4]School of Engineering, Macquarie University, NSW, 2109, Australia

Table S1: Single crystal X-ray diffraction (SCXRD) data:

Compound	$CH_3NH_3PbBr_3$
T/K	300(2)
Crystal system	Cubic
Space Group	*Pm-3m*
a/□	5.9301(1)
b/ □	5.9301(1)
c/ □	5.9301(1)
α/deg	90
β/deg	90
γ/deg	90
Z	1
V/$□^3$	208.54(1)
D_0/g·cm^{-3}	3.989
Reflection collected	9912
Unique reflections	73
R_{int}	0.107
$T_{min.}$, $T_{max.}$	0.176, 0.746
Goodness-of-fit on F^2	1.47
R_1[a)],wR_2[b)][$I>2(I_0)$]	0.060, 0.109
R_1[a)],wR_2[b)] (all data)	0.069, 0.131
$(\Delta\rho)\max, (\Delta\rho)\min/\mathrm{e}\ \mathrm{Å}^{-3}$	2.99. -4.48

a) $R_1 = \sum\left||F_0| - |F_c|\right| / \sum |F_0|$

b) $wR_2 = [\sum w(F_0^2 - F_c^2)^2 / \sum w(F_0^2)^2]^{1/2}$

Table S2: Parameters for Ansys® simulations:

Material	Thermal conductivity ($W\ m^{-1}\ C^{-1}$)	Density ($kg\ m^{-3}$)	Specific Heat ($J\ Kg^{-1}\ C^{-1}$)
$MAPbBr_3$ (a)	0.44 [1]	3830 [2]	356.2 [3]
Copper (b)	400	8933	385

(a) Values for $MAPbBr_3$ were taken from literature. (b) Values for copper were taken from the Ansys® engineering data table.

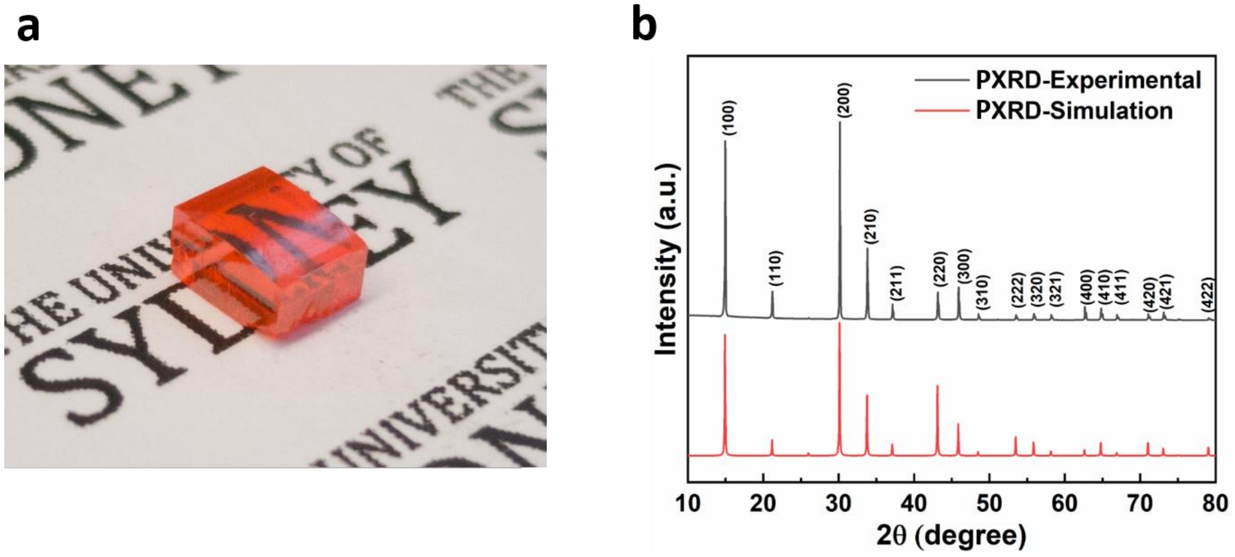

Figure S1: Perovskite growth. (a) Image of a typical crystal grown for experiments. (b) Experimental (top) and simulated (bottom) PXRD patterns of $MAPbBr_3$. The experimental

spectrum was taken from single crystals crushed to form a powder. The simulated spectrum was calculated from single crystal XRD data.

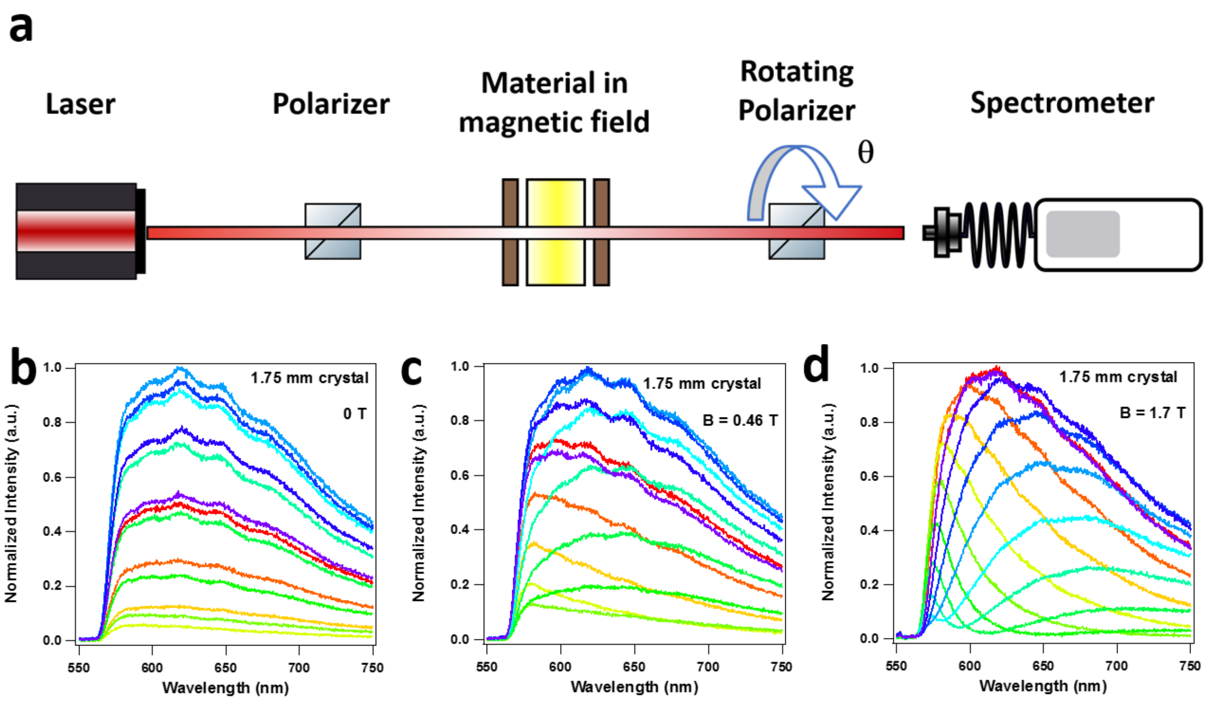

Figure S2: Optical rotation. (a) Experimental setup for Faraday rotation experiments. (b) Transmission spectra of white light probe for a 1.75 mm $MAPbBr_3$ crystal in 0 T magnetic field, at different polarizer angles (θ). (c) Transmission spectra of white light probe for a 1.75 mm $MAPbBr_3$ crystal in 0.46 T magnetic field, at different polarizer angles (θ). (e) Transmission spectra of white light probe for a 1.75 mm $MAPbBr_3$ in 1.7 T magnetic field, at different polarizer angles (θ). Note that only a fraction (1/3) of the total spectra are shown for clarity. From these intensity spectra, selecting a single wavelength and plotting intensity versus angle provides the traces found in Figure 2a-b.

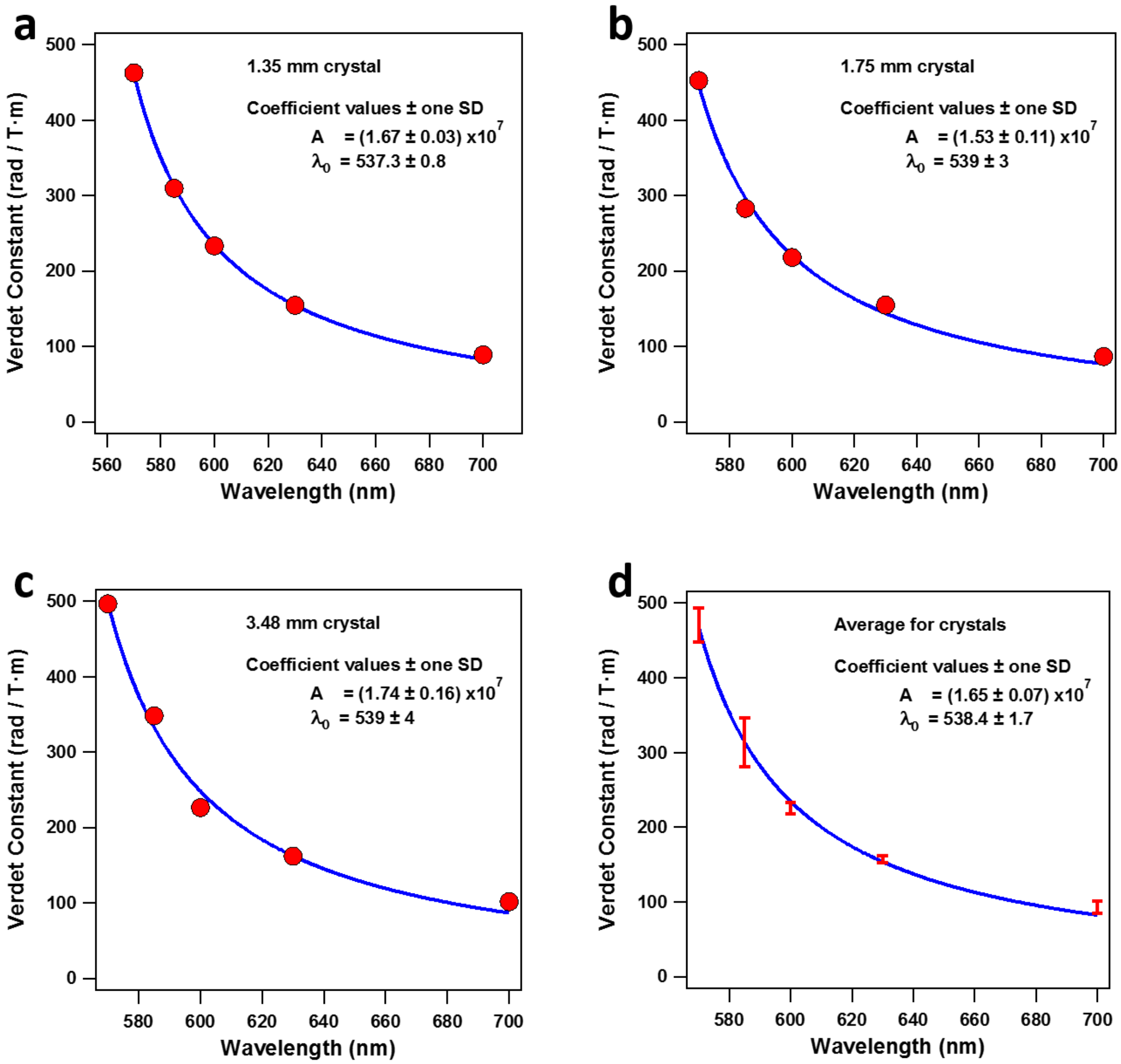

Figure S3: Verdet constants. (a) Wavelength dependence of the Verdet constant for 1.35 mm $MAPbBr_3$ crystal. (b) Wavelength dependence of the Verdet constant for 1.75 mm $MAPbBr_3$ crystal. (c) Wavelength dependence of the Verdet constant for 3.48 mm $MAPbBr_3$ crystal. (d) Wavelength dependence of the average Verdet constant for the three $MAPbBr_3$ crystals, with the associated standard deviations. Coefficients A and λ_0 were obtained by fitting the wavelength dispersion of the Verdet constants. The slope of the Verdet constant for $MAPbBr_3$ is steeper than

that of TGG (Figure S4) because the dispersion becomes less dramatic at wavelengths further from λ_0. The λ_0 of $MAPbBr_3$ is 538 nm, whereas it is 249 nm for TGG. Therefore, $MAPbBr_3$ will have a steeper slope in our wavelength range of interest.

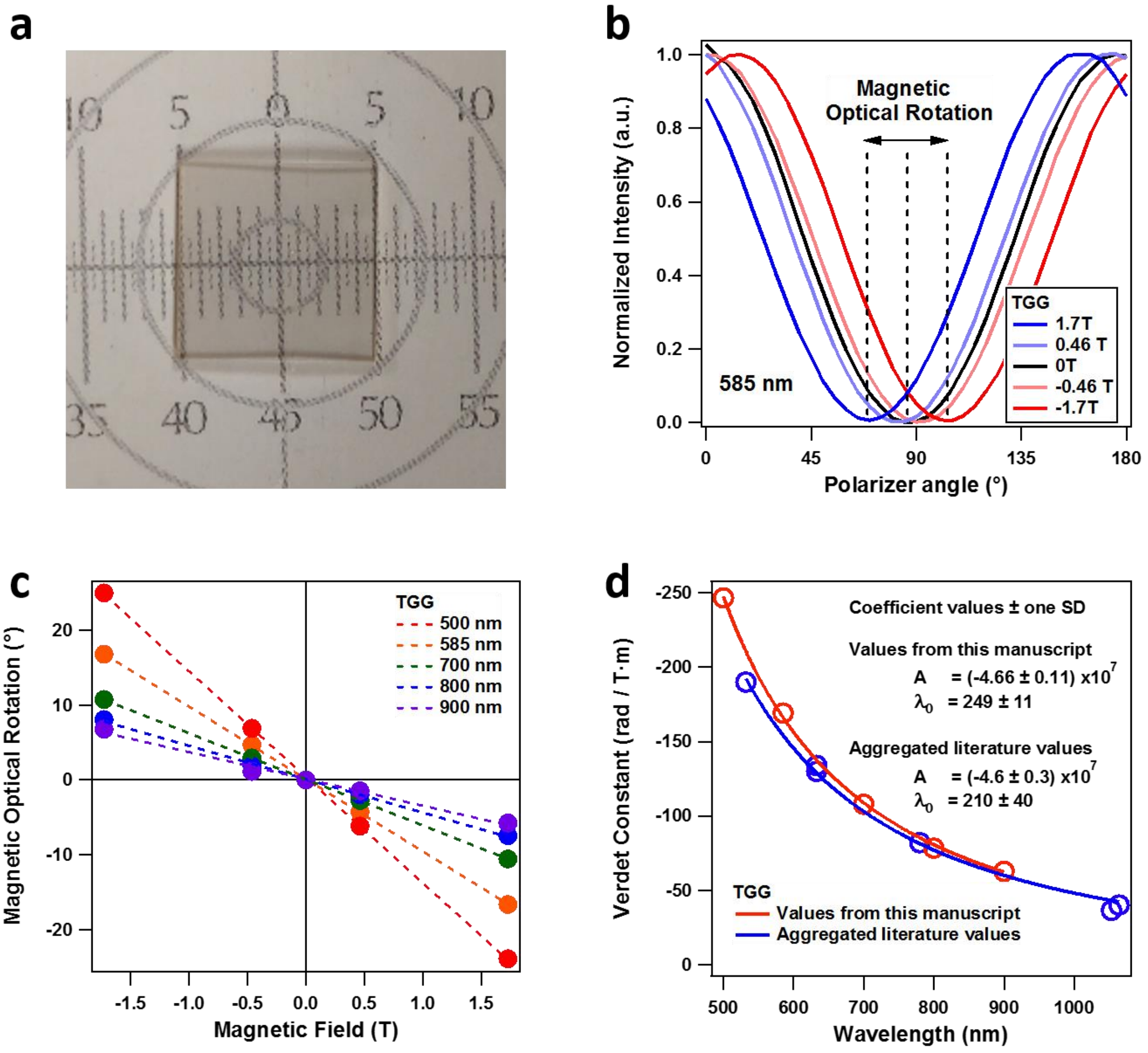

Figure S4: TGG control. (a) Picture of 1 mm thick TGG crystal used as a control. (b) Normalized intensity versus polarizer angle for 585 nm for multiple magnetic fields. (c) MOR of

TGG as a function of magnetic field for different wavelengths. (d) Comparison of Verdet constants of TGG in this manuscript to those observed in the literature.[4-7]

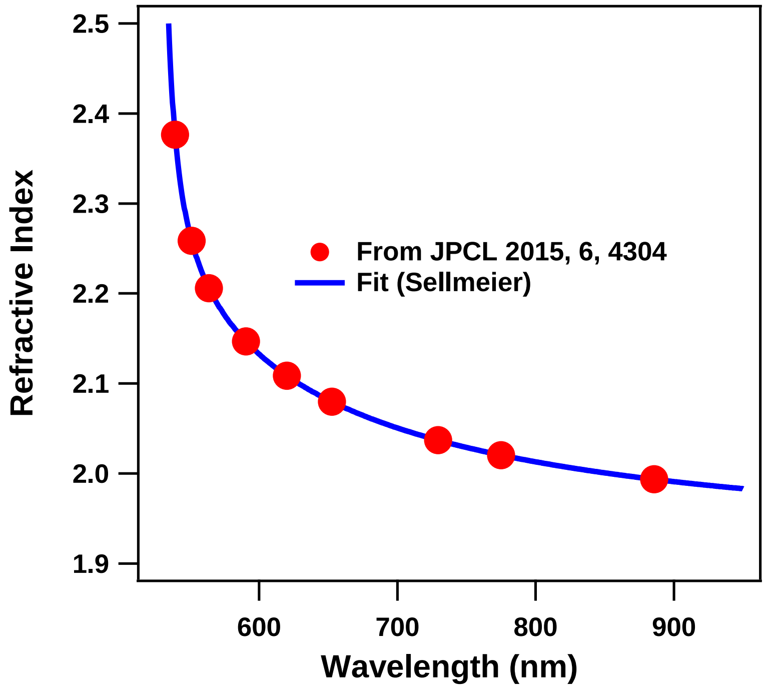

Figure S5: Fitting of literature refractive index values for $MAPbBr_3$ using the Sellmeier equation.

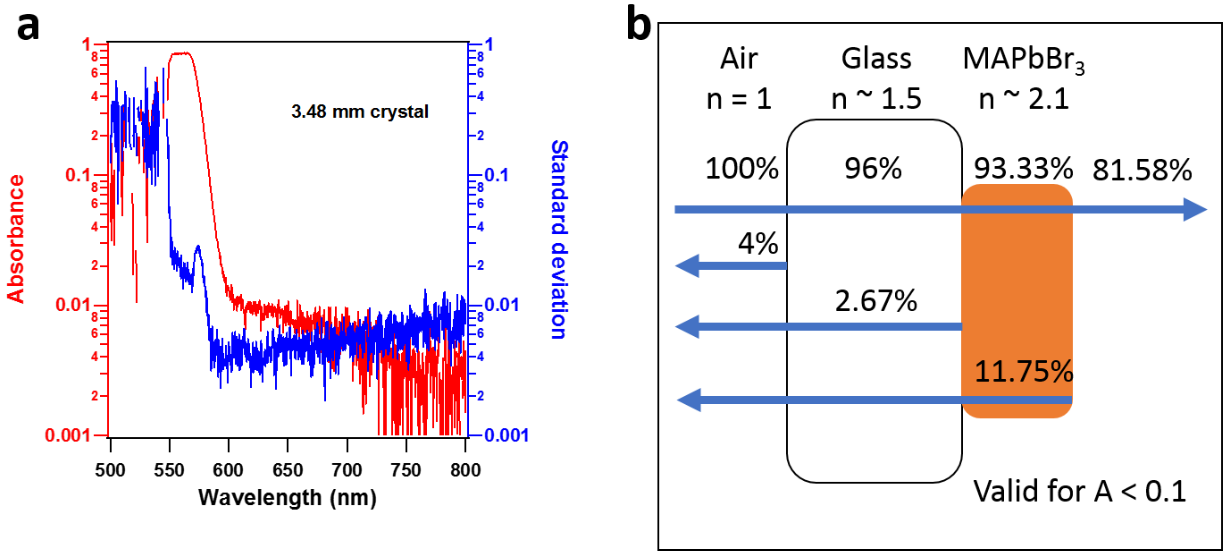

Figure S6. Absorption. (a) Average absorption spectrum (from 9 measurements on same crystal) of sub-bandgap region of $MAPbBr_3$, along with standard deviation. Each measurement for both direct and indirect illumination involved taking the sample out of the integrating sphere and placing it back in (*i.e.* 18 times). Standard deviation in the red end of the spectrum is higher due to less white light probe intensity. (b) A simplified schematic showing the validity of the absorption measurement. Incoming light first hits the air/glass interface, then glass/$MAPbBr_3$. Reflections at both interfaces cause a certain percentage of light to never transmit through the sample. However, at the $MAPbBr_3$ interface, reflected light passes through the sample twice. As the total amount of light traveling through the sample twice outweighs the amount of light never reaching the sample, we calculate that the absorption measurement is valid (*i.e.* if anything, overestimating absorption slightly) for $A < 0.1$. The calculations were carried out for normal incidence, but similar results are expected for other angles.

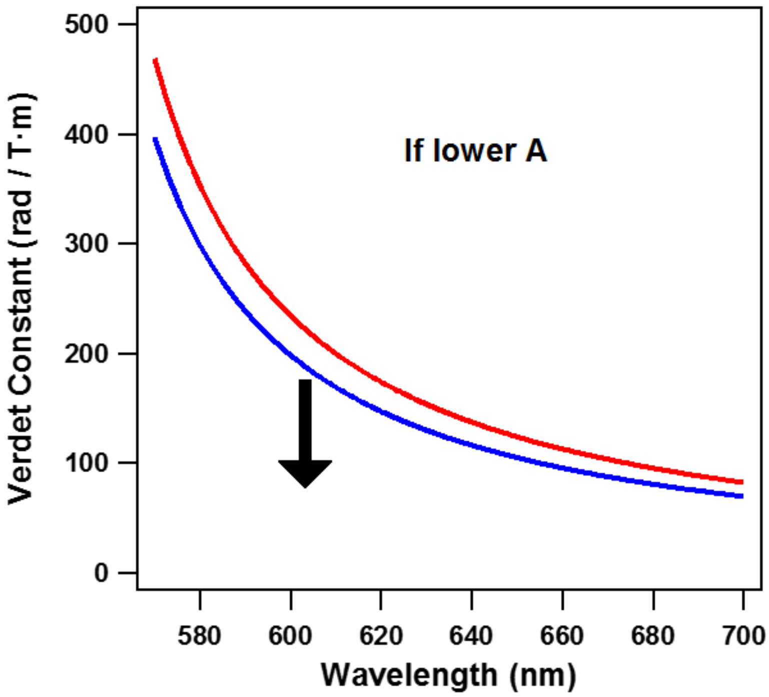

Figure S7: Effect of decreasing A on the Verdet constant according to $V(\lambda) = \frac{A}{\lambda^2 - \lambda_0^2}$. The Verdet constant decreases by the same percentage across the entire spectrum.

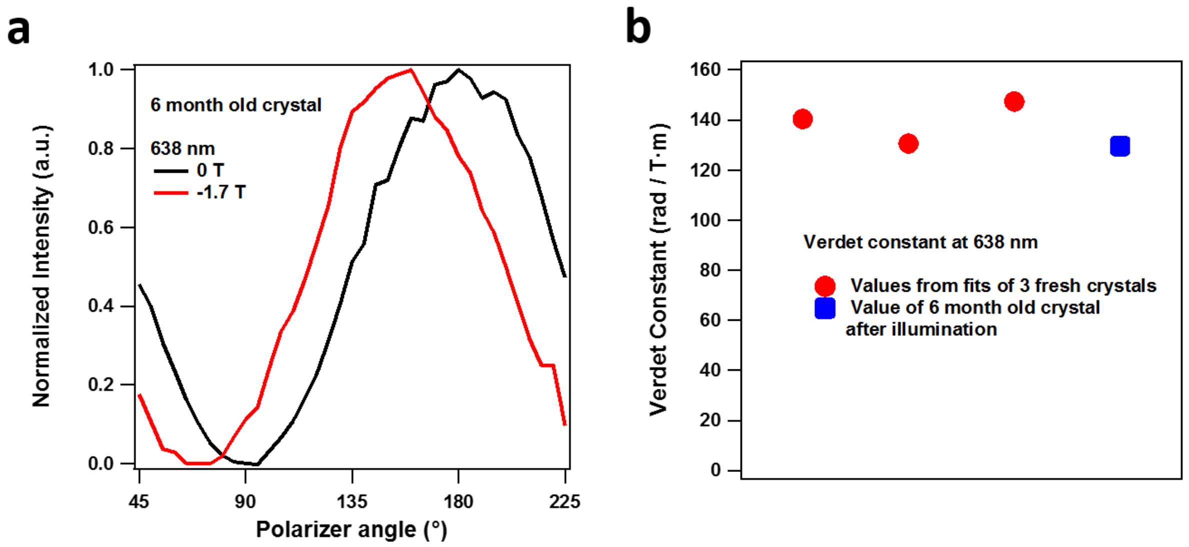

Figure S8: Stability. (a) Normalized transmission intensity versus polarizer angle for a 6-month old crystal of 1.78 mm diameter, in no magnetic field (0T) and a -1.7 T magnetic field. Crystal was illuminated with a 638 nm laser diode at 600 mW/cm^2 for five hours before measurement. (b) Verdet constant after aging in ambient atmosphere and light illumination (638 nm, 600 mW/cm^2, 5 hours), showing negligible difference compared to previously measured fresh crystals.

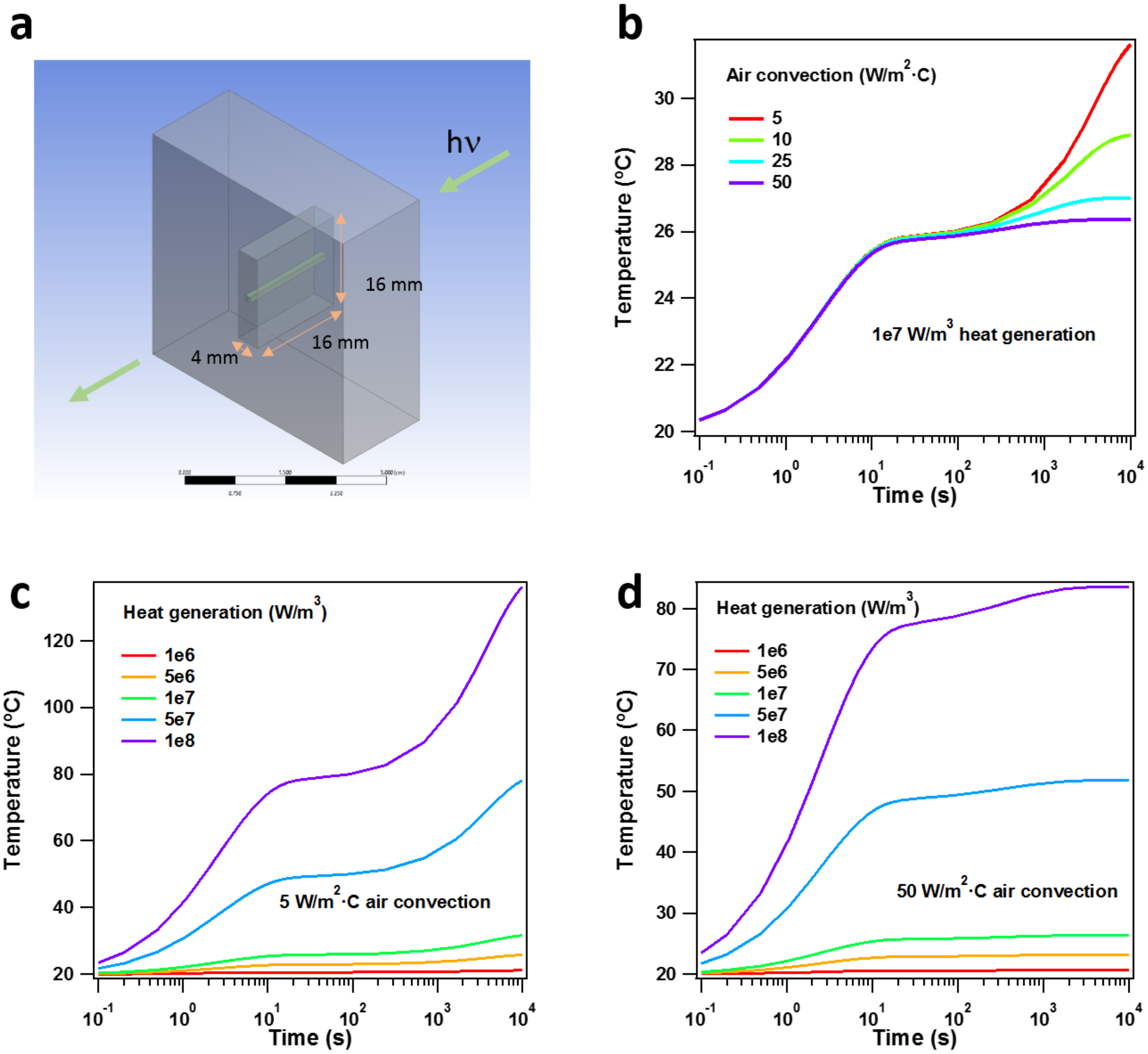

Figure S9: Thermal simulations. (a) Device used in thermal simulations. Note that no appreciable change occurred with the addition of a thin layer of thermal paste (~1-10 μm thick, 1 $Wm^{-1}C^{-1}$ thermal conductivity) between the crystal and copper, and thus the thermal paste was left out of simulations for simplicity (e.g. for 1e7 W/m^3 heat generation and 5 W/m^2 convection, even a 1 mm thick thermal paste layer increased the final temperature by less than one degree). (b) For a given value of heat generation, temperature increase as a function of air convection. (c) For 5 W/m^2C air convection (stagnant air), temperature increase as a function of heat generation/input power. (d) For 50 W/m^2C air convection (forced air), temperature increase as a function of heat generation/input power.

Note S1:

To limit transmissive losses, anti-reflection coatings would be added to the perovskite interfaces. The simplest way to achieve this would be to deposit a transparent dielectric film of material, whose index of refraction is equal to the square root of the index of $MAPbBr_3$. If the thickness of the film is equivalent to a quarter of the desired wavelength, then reflection is theoretically forbidden (and experimentally greatly diminished). For $MAPbBr_3$ at 630 nm operation, its index of refraction is ~2.1, and thus a material with n ~ 1.45 would be desired. Both SiO_2 (n_{630} ~ 1.52) and MgF_2 (n_{630} ~ 1.38) are possible candidates. MgF_2 would be preferred, as it can be thermally evaporated (versus evaporated by e-beam); which we expect to help maintain the structural integrity of the perovskite single crystal. Gold has been thermally evaporated on $MAPbBr_3$ single crystals for photodetector applications,[8] so the crystals should be amenable to this process.

References:

[1] R. Heiderhoff, T. Haeger, N. Pourdavoud, T. Hu, M. Al-Khafaji, A. Mayer, Y. Chen, H.-C. Scheer, T. Riedl, *J. Phys. Chem. C* **2017**, *121*, 28306.

[2] M. R. Leyden, L. Meng, Y. Jiang, L. K. Ono, L. Qiu, E. J. Juarez-Perez, C. Qin, C. Adachi, Y. Qi, *J. Phys. Chem. Lett.* **2017**, *8*, 3193.

[3] N. Onoda-Yamamuro, T. Matsuo, H. Suga, *J. Phys. Chem. Solids* **1990**, *51*, 1383.

[4] R. Yasuhara, S. Tokita, J. Kawanaka, T. Kawashima, H. Kan, H. Yagi, H. Nozawa, T. Yanagitani, Y. Fujimoto, H. Yoshida, M. Nakatsuka, *Opt. Express* **2007**, *15*, 11255.

[5] L. Weller, K. S. Kleinbach, M. A. Zentile, S. Knappe, I. G. Hughes, C. S. Adams, *Opt. Lett.* **2012**, *37*, 3405.

[6] Z. Chen, L. Yang, Y. Hang, X. Wang, *Opt. Mater.* **2015**, *47*, 39.

[7] Z. Chen, L. Yang, Y. Hang, X. Wang, *J. Solid State Chem.* **2016**, *233*, 277.

[8] H. Liu, X. Wei, Z. Zhang, X. Lei, W. Xu, L. Luo, H. Zeng, R. Lu, J. Liu, *J. Phys. Chem. Lett.* **2019**, *10*, 786.
